# The Fine Line: Rural Justice, Public Health and Safety, and the Coronavirus Pandemic

**DOI:** 10.1177/00027642211003144

**Published:** 2021-03-24

**Authors:** Jennifer Sherman, Jennifer Schwartz

**Affiliations:** 1 Washington State University, Pullman, WA, USA

**Keywords:** rural, criminal justice, COVID-19, culture, public health

## Abstract

In this article, we provide an early glimpse into how the issues of public health and safety played out in the rural United States during the coronavirus pandemic, focusing on Washington State. We utilize a combination of news articles and press releases, sheriff’s department Facebook posts, publicly available jail data, courtroom observations, in-depth interviews with those who have been held in rural jails, and interviews with rural law enforcement staff to explore this theme. As elected officials, rural sheriffs are beholden to populations that include many who are suspicious of science, liberal agendas, and anything that might threaten what they see as individual freedom. At the same time, they expect local law enforcement to employ punitive measures to control perceived criminal activity in their communities. These communities are often tightly knit, cohesive, and isolated, with high levels of social support both for community members and local leaders, including sheriffs and law enforcement. This complex social context often puts rural sheriffs and law enforcement officers in difficult positions. Given the multiple cross-pressures that rural justice systems faced in the wake of the COVID-19 pandemic, we explore the circumstances in which they attempted to protect and advocate for the health and safety of both their incarcerated and their nonincarcerated populations. We find that certain characteristics of rural communities both help and hinder local law enforcement in efforts to combat the virus, but these characteristics typically favor informal norms of social control to govern community health. Thus, rural sheriff’s departments repeatedly chose strategies that limited their abilities to protect populations from the disease, in favor of appearing tough on crime and supportive of personal liberty.

## Introduction

In the spring of 2020, the coronavirus pandemic caught most of the United States off guard. Despite warnings that a global pandemic could be devastating and likely, the nation did not have an immediate or well-coordinated response plan in place. Washington State, where the outbreak was first recognized, reacted with more seriousness than many states, particularly those in the South and Southwest. Nonetheless, the state’s size, diversity, and political fissures were liabilities from the early stages of the pandemic and created impediments for controlling the virus. Rural communities across the state faced particularly complex circumstances. As compared with urban settings, rural areas not only enjoyed advantages but also faced challenges when it came to combatting the outbreak. For rural Washington law enforcement, a constant tension between public health and public safety combined with the complex cultural and political climates to complicate their responses in unique, and often underrecognized ways.

In this article, we draw on multiple data sources to provide an early glimpse into the ways in which the rural context interacted with issues of public health and safety during the coronavirus pandemic in Washington State. We utilize a combination of news articles and press releases, sheriff’s department Facebook pages, publicly available jail data, courtroom observations, in-depth interviews with those who have been held in rural jails, and interviews with rural law enforcement staff to explore the experiences of rural Washington’s jails and sheriff’s offices. Rural sheriff’s departments not only patrol vast areas and assist citizens in myriad ways, they also are responsible for administering local jails. Rural sheriffs in Washington are elected officials beholden to populations that include many people who are suspicious of science, liberal agendas, and anything that might threaten what they see as individual freedom, but who also expect local law enforcement to be tough on crime and to focus on punitively controlling certain types or categories of crime. These communities are often tightly knit, cohesive, and isolated, with high levels of social support for community members and local leaders, including sheriffs and law enforcement. This complex social context puts rural sheriffs in difficult positions, heavily constraining their abilities to react to the pandemic. We explore the multiple pressures that rural justice systems faced in the wake of the COVID-19 pandemic, and the contradictory responses that challenged their abilities to fully protect or advocate for the health and safety of both their incarcerated and their nonincarcerated populations. We find the rural context to be both help and hindrance to combatting the virus in Washington State, not only providing some protection from rapid spread but also often constraining sheriffs to informal methods of social control where public health is concerned.

## Literature Review: The Coronavirus Pandemic, Rurality, and Criminal Justice

Despite potentially protective factors like low density and distance to urban epicenters, it did not take long for the coronavirus pandemic to spread to rural America (Babwin & Weber, 2020; Leatherby, 2020; Orgera et al., 2020). Correctional facilities were among the first hot spots of virus spread in rural communities, along with meat and fruit packing plants and Native American reservations (Babwin & Weber, 2020; Martin, 2020; Rodriguez-Lonebear et al., 2020; Schafft & Maselli, in press; Thebault & Hauslohner, 2020). Many rural communities already suffered from high levels of social vulnerability,^1^ intense crowding in correctional facilities (Kang-Brown & Subramanian, 2017; Ruddell & Mays, 2007), and a lack of personal protective equipment (PPE) and health care providers and facilities. In addition, rural populations are often older, poorer, and less healthy than those residing in urban and suburban areas (Monnat, 2020; Peters, 2020; Thebault & Hauslohner, 2020).

The combination of older and vulnerable populations with lack of medical facilities and providers were factors that increased concerns about the destructive rural potential of COVID-19. These health concerns were exacerbated by many communities’ proximity to high-density facilities including nursing homes and correctional institutions (Orgera et al., 2020; Peters, 2020), which could further put strain on medical infrastructure. As Monnat (2020) explains,

Even when hospitals are available in rural areas, they do not have the capacity to deal with a surge in coronavirus cases, and they have limited availability of medical personnel, ventilators, personal protective equipment (e.g., masks, gloves, gowns), and other essential supplies. Particularly concerning is that only 1% of the nation’s ICU beds are located in rural areas. In addition, rural physicians are older than their urban counterparts, meaning that they are at very high risk while providing services.

In this context, rural criminal justice systems faced major dilemmas. Both prisons and jails were quickly branded as “petri dishes” that provided nightmare scenarios in terms of virus transmission (Williams et al., 2020; Yan, 2020). Concerns included spread among the incarcerated population and among staff, who could then potentially bring the disease into communities, resulting in significant threat of sickness and death (Lofgren et al., 2020; Oh & Abrams, 2020). Jails present a particularly dire challenge in that they are generally housing people for short-term stays, resulting in high turnover and constant mixing between the nonincarcerated and incarcerated populations. In addition to often being located in communities that lack health care infrastructure, rural jails present other dangers. People in jail are often in poor health due to factors including age, diet, and long-term drug and alcohol use (Kang-Brown & Heiss, 2020; Littman et al., 2020). Furthermore, rural America contains “some of the nation’s most overcrowded jails . . . where close quarters and poor sanitary conditions are a recipe for disaster” (Kang-Brown & Heiss, 2020). The combination of health concerns, crowded facilities, high turnover, and lack of medical infrastructure has made rural jails a major concern for both communities and incarcerated populations (Goodell, 2020; Littman et al., 2020; Oh & Abrams, 2020).

As with urban jails, there were limited options in order to control the spread of the disease both within jails and from jails to the surrounding communities. Experts recommended structural reforms for criminal justice institutions such as reducing inmate populations through decreased bookings and increased/early releases. A study by Lofgren et al. (2020, p. 16) found:

If jurisdictions across the country reduce their intake by significant percentages, our models demonstrate that we will meaningfully directly reduce the disease incidence in the incarcerated population . . . Moreover, these same strategies also clearly produced a reduction in the source of risk to incarcerated people’s families, jail staff, and the broader community. These strategies could be enacted in a number of ways, such as (but not limited to) replacing misdemeanor arrests with citations, avoiding recommendations for jail time or prohibitive terms for bail conditions, or refusing to detain anyone for nonpayment of fines or fees during the course of the outbreak.

While most jurisdictions have taken steps in these directions, for many conservative rural residents in particular the idea of decarceration can be a hard sell. Although it makes sense in terms of securing the health of the community, for many rural residents “safety” is seen as separate from health rather than intertwined, and toughness on crime is a virtue (Weisheit et al., 1994). Such cultural contexts create challenges for rural sheriffs, who as elected officials cannot easily afford to ignore the will of local populations.

Although rural communities are not monolithic, the tendency toward conservatism has been documented in rural America for decades (Dillon & Henly, 2008; Frank, 2004; Kelly & Lobao, 2019; Knoke & Henry, 1977; Sherman, 2009), and particularly since the election of Donald Trump in which he carried more than 60% of the rural vote (Kurtzleben, 2016; Morin, 2016). Recent research includes both qualitative and quantitative evidence of the links between rurality and conservative cultural norms and voting trends, including racism, resentment, and reactionism (Ashwood, 2018; Cramer, 2016; Hochschild, 2016; Monnat & Brown, 2017; Sherman, 2021; Ulrich-Schad & Duncan, 2018; Wuthnow, 2018). Researchers paint a picture of rural America as politically more conservative than many urban settings, and more likely to be concerned with issues like gun control, immigration, and individual liberty than social welfare or health. For those working in rural law enforcement, this cultural context can mean they walk a fine line between public health and safety and retention of community support, particularly in the context of the coronavirus and the questions circulating regarding the science and true nature of the health threat.

This article will explore this tension, and the double-edged sword of rurality in the age of COVID-19. While many rural sheriffs and those who work alongside them have focused on the virtues of small and cohesive communities, including their relative isolation, support for fellow citizens, and initial sense of physical distance from the virus, they nonetheless also ultimately confront the challenges discussed above. However, they do so somewhat warily, in cultural contexts that are often hostile to many of the very measures that are most helpful in slowing the spread of the virus, including limits on freedom of movement and congregation, mandates to cover one’s face in public, and easing of incarceration for less-serious crimes. Through our in-depth exploration of rural Washington criminal justice and its trajectory in the first 7 months of the pandemic, we argue that rural communities are particularly vulnerable during the crisis due to a combination of lack of resources and infrastructure in a cultural context that is often hostile to measures that may be interpreted as threatening to individual freedom and agency.

## Research Methods

This article presents preliminary results from an ongoing study of rural jails and justice in Washington State, with a focus on its more socially conservative Central and Eastern regions. The research began in early 2020, just as the pandemic was beginning, and uses multiple methods and angles to assess the drivers and impacts of rural jail incarceration. Among the sources of data for this article include both informal and formal interviews with rural sheriffs and department personnel, ongoing in-depth interviews with individuals who have recently stayed in rural Washington jails, quantitative data from county jail rosters and booking logs, ethnographic observation of (online) courtrooms, and ongoing analysis of rural sheriffs’ Facebook posts and press releases.^2^ Together these sources of data allow us to provide a multifaceted—albeit, early—picture of the multiple pressures, advantages, and struggles faced by rural Washington sheriffs, counties, and communities in their attempts to control the coronavirus pandemic.

The quantitative analysis for this project come from daily jail registers made available to the public under state law.^3^ Jail records in the form of booking logs and/or inmate rosters provided information on daily populations, new admissions and causes of jail confinement, and releases from jails in rural Eastern and Central Washington counties. We began collecting data on March 16, 2020, about 2 weeks after the first coronavirus case was identified in Washington and before widespread stay-at-home orders took effect. Central Washington counties had reported their first coronavirus cases, but none had yet been reported in rural Eastern Washington when we began data collection. We report results through mid-August, although data collection is ongoing.

The qualitative analysis for this project includes multiple types and sources of data that are currently being collected and analyzed. Among our qualitative data sources are a number of informal and formal conversations and interviews with justice staff in which both authors took part, both before the pandemic began and once it was underway. The larger research project includes a partnership with multiple rural jurisdictions,^4^ and it is with these partners that we have had ongoing conversations about their contexts, concerns, and responses to the pandemic. While these conversations have been mostly unstructured, they nonetheless have provided important information regarding the issues faced by rural Washington sheriff’s departments, as well as their strategies for responding to the coronavirus pandemic.

Jennifer Sherman has also been conducting in-depth, semistructured, recorded phone interviews with individuals who have been detained at least once in a rural Washington county jail. Due to the pandemic, our ability to conduct interviews with those currently incarcerated has been temporarily curtailed, as safety precautions preclude us from entering jails and privacy concerns regarding confidentiality inhibit phone calls from inside jails. For the duration of the pandemic, the research is thus focusing on those who have been recently held in (and subsequently released from) rural jails. Recruitment has occurred through multiple sources, including classified ads in rural newspapers and craigslist boards, posts in justice-related and rural community Facebook groups, snowball sampling and word of mouth, fliers handed out by jail staff on release, and fliers posted in social service agencies and public locations such as post offices and libraries throughout rural Eastern and Central Washington and into Northwestern Idaho. All participants are offered $25 gift cards as incentives, which are delivered via email, text, or mail depending on their preferences. Interviews include questions focused on participants’ background and social networks, criminal justice involvement (we do not press for details about specific crimes or cases), the coronavirus pandemic and health concerns, demographic information and safety-net usage, and a series of questions that allow participants to reflect on their lives and choices. To date, 25 interviews have been completed, including seven whose jail stays occurred during the pandemic, providing us with some preliminary insight into the experiences and concerns of the rural justice-involved during this timeframe.

During the pandemic, the research team has also been conducting ongoing ethnographic observation of virtual courtroom activities in multiple rural Washington counties. As will be discussed more below, since the pandemic the bulk of Washington courthouses have been closed to in-person appearances, and thus hearings and trials have been conducted online. Several of the rural counties included in this study have made their virtual courtrooms available to the public, facilitating this observation. Approximately 12 hours per week of observations have been completed for 6 months thus far, with data collection ongoing. Data are collected through extensive notetaking of these observations.

Finally, the research team have also collected and recorded public posts from the Facebook pages of sheriff’s departments across rural Washington, beginning in March and continuing throughout the pandemic, including those of the six jurisdictions in our partner network. We look in depth at both the official posts and the number and content of reactions and comments to the posts, in order to determine both the messaging and public foci of rural sheriffs and the ways in which these messages are received by their constituent communities. Although the posts may not reflect the full picture of rural law enforcement’s concerns or strategies, we interpret these posts as representing their attempts to manage their public faces and communicate directly with local populations. We similarly recognize that the reactions they receive do not represent a full or unbiased picture of community reactions but nonetheless do provide insight into the most vocal and vehement responses that law enforcement receives. Thus, there is much insight that can be gained from these posts regarding how these departments interact with and respond to the public and its preferences with regard to the pandemic. Although this work is ongoing, for the purpose of this article, we have conducted preliminary analyses of the qualitative data in NVivo software, creating a coding scheme that allows for identifying and analyzing multiple themes across our data and variation within them.

## Results: Rural Criminal Justice, Health, and Managing Community Responses

### Early Responses to COVID-19: Balancing Community Health and Public Safety

Washington State issued one of the nation’s earliest stay-at-home orders, which in late March closed many businesses and requested that individuals shelter at home and limit trips for any purpose beyond basic needs and essential work. While such orders have since proven to be quite effective at slowing the virus spread (Castillo et al., 2020; Fowler et al., 2020), from the outset law enforcement struggled with how to implement what turned out to be an extremely controversial edict. The stay-at-home order was particularly unpopular in rural areas, many of which did not see local cases for several months and had populations who resist perceived impositions on personal freedoms and liberty. Sheriffs told us in interviews that it was difficult to enforce safety measures when “you’ve got a community that aren’t seeing any of what’s being talked about.” Facing strong backlash from constituents who questioned the reality and/or severity of the pandemic, many jurisdictions emphatically assured the public that the orders would not be actively enforced and did not amount to “martial law.” They used the language of recommendation, education, and encouragement rather than law and order tactics against citizens, as in the following Facebook examples:

The County Sheriff’s Office [is] strongly encouraging community members to stay at home when not performing a necessary or essential function. We understand the health of the community and rights of individual citizens must be appropriately balanced, which is why we are asking for compliance and self-regulation for the next two weeks.

Please stay at home if you have that option. Limit contact with others and honor the requests and recommendations of your local, state and national leaders. (March 24, 2020)

Law enforcement’s primary role is to help educate people about how to comply with orders to not gather together, to stay at home, and other restrictions. Law enforcement is not being asked to detain, arrest, ticket or establish checkpoints for compliance. Rumors of strict law enforcement or “martial law” are simply not true. (Posted in coordination by multiple county sheriffs on March 24, 2020 and March 25, 2020)

Under the order of the President, Governor and local health officials, we strongly encourage social distancing.

As other law enforcement officials have already stated, we will not be making arrests or issuing citations for gathering but rather trying to educate the public. (March 25, 2020)

While such statements were clearly meant to placate opposition to the stay-at-home orders, they were often frustrating to individuals who *were* taking the orders seriously. Rural sheriffs made repeated attempts to assure the public that they would not be patrolling and making arrests for violations of the stay-at-home order, but a number of individuals complained publicly that they should be enforcing the order more strictly. The Washington Association of Sheriffs and Police Chiefs acknowledged this conflict in a May 2020 statement: “Understandably, some are frustrated by people violating and not having a consequence, while others are frustrated by the order and its disruptions.” Sheriffs’ Facebook pages reflected this, with some commenters protesting the orders, and others complaining about “drug addicts” and “methheads” not practicing social distancing and urging local law enforcement to ticket those who disobeyed the orders (April 4, 2020; April 6, 2020). One commenter asked, “Do you think you all could drive through town and remind people of the importance of staying home? . . . It’s not a vacation or shopping spree, it’s for essentials . . . not every day trips to town.” (April1, 2020).^5^ Many focused on outsiders entering their communities for outdoor recreation and called for targeted enforcement of these out-of-towner violations. Difficulties in finding the balance between “tyranny” and nonenforcement weakened the effectiveness of the statewide order, particularly in rural parts of the state.

The stay-at-home order was replaced in early summer with limited phased reopenings, which progressed more quickly in rural areas that had seen few cases at first. As cases then began to rise in both rural and smaller urban areas across the state, the Department of Health issued new orders in late June, requiring masks in public spaces, workplaces, and businesses. For rural law enforcement, similar issues undermined the effectiveness of these orders, and once again rural sheriffs made clear that they would not actively enforce them. One sheriff in our study explained in an interview:

Particularly in a very rural area where our main street might have, what, five people in six blocks walking at the same time, the blowback from the community—and the cooperation from the community would’ve absolutely been sacrificed if I would’a done it . . . and I just decided that we’re not going to enforce that.

He went on to assert, “This is the best call in order to ensure cooperation from the public.” Most of the rural sheriffs in our study took similar stances, and many made public statements like this one, from a July 6 Facebook post: “Considering that [the] County has not had a single positive test since shortly after the shutdown started, the enforcement of this order will not be a priority and therefore will not be enforced.” On that same day, another sheriff posted, “We will not be taking criminal action with individuals who choose not to wear the mask. We will try to educate but we will not take criminal action [on the mask ordinance].” Several neighboring counties jointly released a statement on June 24 that pledged inaction on the order, saying, “We will continue to focus on crimes and criminals which impact public safety. The statewide face covering order is a public health and safety measure, it is not a mandate for local law enforcement response.” Rural sheriffs repeatedly drew a line between public health and public safety, insisting that their jurisdiction only included the safety side. With regard to health, it was up to individuals to protect themselves, and informal responses like education and encouragement were all they would provide.

Public response to these statements was largely in agreement that rural constituents did not want local law enforcement to police their mask-wearing behaviors, with harsh words often for those who did follow the orders. These included the residents who commented on Facebook in late June, “Thank you! The freedom to choose whether you would like to or not,” “Glad to live in a County that uses common sense! 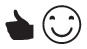 Not a sheep!” and “Thank you for putting crime and safety above mask wearing or not.” The contrast between freedom and common sense versus tyranny and blind rule following was highlighted by many of the posters who supported nonenforcement, which was the great majority of those who commented on sheriffs’ posts. Many suggested that the mask order was a direct assault on their constitutional rights, including this poster who saw it as a freedom of speech issue, “Well I don’t plan on wearing mine because of First Amendment.” Others conflated the mask ordinance with dictatorships and communism:

Its a personal choice. If I’m sick I stay home, I’m not rioting, I’m not invading peoples personal space. If you have health risks wear a mask. I would rather get the Wuhan virus and get the antibodies then run around scared. I will not wear a mask they can cite me if they choose. You do you, I’ll do me. Last I checked the communists have not taken over yet and this is still a free country.

Although there were also posters who expressed a desire for enforcement of the mask ordinance, as well as some who supported the use of masks without supporting enforcement, by and large on this issue rural sheriffs followed the sentiment of the bulk of their more visible constituents and backed away from enforcing the orders, allowing sporadic and inconsistent use of masks throughout many rural Washington communities.

In addition to trying to maintain health through behavioral modifications like these, rural sheriffs were also faced with conundrums regarding how to address crime and legal issues during the outbreak. Early in the pandemic, it became clear that incarceration facilities had the potential to spread the virus quickly. Thus, as in urban areas, for rural jurisdictions a first line of defense was to reduce jail populations. The jails in our study all reduced their incarcerated populations and undertook additional safety measures meant to slow the spread within jails and between jails and the larger communities. Rural jails that had been operating near or beyond maximum capacity reduced their populations by as much as half, allowing segregation of the newly confined in separate cells. According to our analyses of booking data and inmate rosters, the population across five of the study’s county jails dropped by more than 40%, from more than 500 people to fewer than 300 over a period of just 3 weeks starting in mid-March. After this initial response, however, by the end of April jail populations again started to slowly rise. By 5 months into the pandemic, the regional jail population had rebounded to about 350 people, still well below the prepandemic level but trending upward.

Rather than large scale releases of those awaiting trial on nonviolent charges, as reported elsewhere (Quinlan, 2020), the main mechanism by which rural jail populations were reduced in this region was via fewer new admissions for (mostly) short term jail stays. Jail admissions slowed markedly, from 15 or more per day in mid-March to just 5 per day across the entire rural region in early April until the end of April. New admissions were averaging 10 per day as of mid-August. Integral to these reductions, the Washington State Supreme Court (March 20, 2020) ordered all courts statewide to cease issuing bench warrants for those failing to appear for court/pretrial hearings. This had an immediate impact in some jurisdictions where, prior to the pandemic, roughly one quarter of the jail population was there due to a bench warrant for failing to appear for appointed pretrial dates. To make further reductions, sheriff departments put in place booking restrictions to divert minor incidents and were less active in seeking out crime (e.g., drug busts/stings). For example, planned, multicounty drug enforcement operations mostly ceased. Nonetheless, when officers detected illicit drugs or paraphernalia during traffic stops, punitive responses still resulted in new bookings. Drugs, along with felonies and mandatory domestic violence arrests, remained consistent enforcement priorities according to jail data. As such, new admissions for these offenses contributed to rural jail populations (and turnover) throughout the pandemic.

One unique component that buoyed rural jail populations was the rather significant share of people being held locally for federal or other agencies. Rural jurisdictions have long relied on federal contracts for housing inmates to supplement lean budgets, and this obligation persisted with little change in many counties throughout the pandemic. Agreements among local jurisdictions to transfer those held on out-of-county warrants were halted, but contracts continued with agencies like the U.S. Marshals Service, responsible for transporting and housing federal prisoners including for immigration-related detentions. In some jurisdictions, one quarter or more of people in jail were detained on behalf of federal authorities.

Beyond limiting new bookings, many rural jurisdictions did also attempt to ensure safety within their jails.^6^ They focused on providing PPE for their staffs and provided training on health and safety protocols. They tried to provide testing to jail populations and to jail staff. The newly confined were quarantined for up to 2 weeks, screened for virus symptoms, and given sanitation instructions. Some were provided with masks. And most counties closed what were deemed nonessential justice services for several months. Forms and applications went online, as did courtroom proceedings and probation checks. Jail visitations, jury trials and fingerprinting services were temporarily suspended in most counties. Together, these measures were expected to be relatively noncontroversial and practical steps to reduce the danger of virus transmission.

However, public response to these sorts of precautionary measures was largely unsupportive in rural Eastern and Central Washington. Regarding reductions in jail populations, residents expressed fear of crime that seemed to be greater than their fear of the virus. Many explicitly or implicitly suggested that the deemphasis on incarceration was directly responsible for criminal activity, including the Facebook users who reacted to a May shooting incident by commenting, “Wow I wonder if it was one they released from jail? Over the covid BS”; “Let’s release more inmates . . . . what a injustice to law abiding citizens!!!” and “You can thank the lovely governor of Washington. He only cares about the druggies and the criminals obviously.”

An earlier post regarding vehicle prowls in the same county elicited a similar response:

Maybe if the Judges in this state would start jailing thieves instead of catch and release this problem would solve itself. This policy is putting police and the public at risk! Judges do your job and start locking up criminals!

Over time, posts became increasingly hostile to law enforcement, and while commenters still congratulated local sheriff’s departments on successfully catching those who committed crimes, several expressed increasing frustrations with the hands-off strategies during the pandemic. The following post from early May exemplifies this anger:

Bunch of pansies [out] here who don’t think criminals should be shot. Leave Spokane [and] try Seattle, it’s full of bleeding heart liberals like yourselves. Trust me us native eastern Washingtonians don’t want you and your dumbass slap the criminal on the wrist and let em go agenda here. We will push you out we always have always will.

These posts made clear that Eastern and Central Washingtonians saw themselves as separate from the rest of the state, and if rural sheriffs wanted to retain their approval it was necessary to demonstrate that they were in solidarity with their communities rather than the more liberal (and urban) West Side of Washington. Moreover, these posts expressed a clear preference for prioritizing “tough on crime” approaches focused on punishing “druggies” and “criminals” to protect “law abiding” citizens and safeguard their personal freedoms. By early summer, the same trend appeared on all the sheriffs’ Facebook pages: decreasing information related to the virus and pandemic, and increasing focus on crime fighting, toughness, and catching suspects.

Regarding the temporary closure of in-person services including fingerprinting there was also a strong backlash expressed by some in rural communities. Generally, these complaints focused on the restriction in issuing new gun permits, which require fingerprinting. Reactions included the following comments:

And it starts . . . government control. The county isn’t doing finger printing for concealed carry for now. So people can’t get ccw to carry guns concealed. Just another way to get weapons out of the hands of non criminals. There is a virus for sure but it [is] definitely being used for political gain!!! What a shame!!!!

Sheriff, I respect you and have voted for you in the past. This is tyranny. Uphold your oath and the Constitution you promised to protect. Do the right thing.

For rural sheriffs, taking precautionary and safety measures was thus neither easy nor simple. While most were attempting to follow both the state regulations and the advice of experts, they faced strong opposition from constituents who saw health and safety measures as a direct threat to their personal freedoms and the safety of their communities. Sheriffs were forced to walk a very fine line between the demands of their local resident populations and the recommendations of researchers and medical experts. Most balanced these competing concerns by choosing not to enforce unpopular health orders, eventually reopening services like gun permitting, slowly increasing arrests and jail populations, and refocusing their messaging on law and order versus the pandemic and related safety concerns.

### Controlling COVID-19 in the Justice System: Impacts of Management Strategies

For those who were involved in the justice system during the pandemic, health and safety measures were both too little and too much; while some complained of inadequate protection from the virus, in other cases protective measures such as extended periods in solitary confinement were difficult and damaging to mental health. In one case, an interview participant described no unusual protective measures beyond being provided with a mask, which he said none of the residents wore. In other jails that took more serious precautionary measures, they often imposed unforeseen hardships on those who were involved in the system, while still not providing sufficient protection against the virus. Among the concerns that arose most frequently in the in-depth interviews were difficulties in accessing remote court dates due to insufficient phone access, protracted waits for trials and hearings resulting in lengthy jail stays and long waits to have charges dropped, and the overly punitive nature of jail quarantine periods meant to isolate the newly confined from the general population in order to curtail the virus spread.

During the pandemic, all the counties in our study moved court appearances to virtual formats, with defendants generally expected to call in via phone or video. Jury trials were suspended, with concerns about how counties would deal with the backlog of cases once they were reinstated. Although virtual court appearances allowed all parties to limit their exposure to the virus, they were not without their problems. Throughout the ethnographic data gathered from watching courtroom proceedings were numerous instances of released defendants failing to appear because of lack of functioning communication equipment.^7^ In several cases, defense attorneys explained that they did not have working phone numbers for their clients and had not had contact with them since the pandemic began. Some people without reliable phones failed to appear; in other instances, phones died in the middle of proceedings. Generally, warrants were not issued for these no-shows, but nonetheless lack of reliable phone or video undercut the justice process for many of the most vulnerable defendants and likely would create additional complications for them further down the line.

In addition to the complications imposed by virtual courts, for those individuals who were taken into custody during the pandemic, safety precautions were often overly punitive. Normally a technique used as punishment, many were held in solitary confinement for the duration of pandemic jail stays. For 56-year-old interview participant Michelle Sanchez,^8^ solitary was a mixed blessing:

When they picked me up one of the times for the failure to appear, like I said, I had just—I had been sick and I don’t know what I had but it wasn’t contagious or anything, but they put me in an isolation cell, which I really liked. Okay? It was an isolation room. I mean, it was all concrete, yeah. No windows or anything. But I had my own little concrete bed and I was there in my—I was by myself. And it was—it was nice. I mean, because I wasn’t there having to deal with other people. I didn’t have to climb down anything. And I could just sit there and read. But I mean, would I recommend that to anybody? Oh, heck, no.

Michelle described herself as an introvert who preferred to spend her jail time alone with a book. However, for others solitary confinement was a hardship that they found personally destabilizing. For 46-year-old Christy Jones, a similar experience was described as significantly more difficult. In her interview, she also described a lack of PPE and sanitary supplies while in solitary confinement:

Because of COVID, they had me in a cell by myself because they had to quarantine anybody who came in or out for two [weeks]—yeah. So I was in a cell all by myself, um, for three days . . . No shower, nothing. Just a cell with a toilet in it . . .

Q: Did they give you soap? Did they give you PPE, you know, masks or anything like that? Gloves?

They gave me a toothbrush and toothpaste and that was all they gave me . . . No, mask, nothing . . .

Q: And what did you do with the time?

I slept . . . Yeah, I slept for . . . .yeah, that’s all I could do. They never brought me the book I asked for, you know? So it was like, okay, I’m just going to sleep as much as I can and hopefully get out soon.

Christy described the experience as “horrible” and undermining her life and mental health.

For many, the pandemic meant considerably harsher punishment for minor crimes. Fifty-four-year-old Karl Bell described being quarantined in solitary confinement in a prison cell, when under normal circumstances he would have been held in a county jail for his probation violation. However, the county jail was not accepting Department of Corrections transfers because of the virus. Karl explained that he did not have a felony, and that he was placed in prison only because of the pandemic and resulting need to reduce jail populations. He described the experience:

It was, like, terrible and quiet. You get locked up 24 hours a day and you have a 15 × 7 cell, and you have got a little window to look at and it’s really boring, and you are boxed up like an animal, and then when you get out in the yard, you go into another cage, 15 × 8, and you get one hour out every other day, and you are locked up 23, 24 hours a day. And in a way, it’s probably good because you are not around people, but you are in max. And 21 days is, like, quite an experience. I wouldn’t want to do it again . . . It was terrible to be locked up like that.

Such measures were meant to protect the health of those incarcerated and control the virus spread within facilities that were simply not constructed for social distancing and controlling disease. However, those incarcerated during the pandemic often experienced jails’ protective measures as both inadequate and overly punitive. The experience of solitary confinement created additional suffering for individuals who often discovered upon release that jobs, housing, and other types of support were even scarcer than before the pandemic. For many jailed during the pandemic, it seemed that public health came at the price of their own mental health as well as personal liberties.

### Rural Vulnerability Versus Exceptionalism

The pandemic has been a difficult time for all of Washington State and especially so for rural communities, which have faced many of the challenges that researchers warned of, including poverty, aging populations, inadequate Internet access, and lack of medical infrastructure. Many also struggled with limited labor markets and economies that were less resilient to measures like the stay-at-home orders.^9^ Most saw their unemployment rates skyrocket during a period when the communities had no confirmed cases of the disease, as in a remote county whose “citizens are still suffering from regulations designed for the masses in urban Washington” (Quinlan, 2020). In counties that were heavily dependent on tourism or that had retail amenities, fears rose that recreators visiting from more crowded and urban areas of the state would bring the virus into communities that were otherwise shielded from it. These and numerous other challenges suggested that the pandemic could be disastrous in rural Washington.

As the previous sections have illustrated, rural Washington sheriffs walked a fine line during the pandemic, delicately balancing between public safety and public health in ways that their communities accepted as valid. As one sheriff explained in an interview, “Law enforcement in general is all about the public trust. And if we do things that erode that trust then we lose the war, not a battle.” Rural constituents often did not see public health and safety as intertwined, and it was easy for sheriffs to quickly lose the trust and support of their local populations for words or deeds that could be interpreted as either being weak on crime or infringing on the personal liberties and freedoms of noncriminals. This context could be very limiting for rural sheriffs and leaders in their attempts to navigate the pandemic, particularly given their structural challenges. As we have illustrated, they often responded by directing efforts at controlling criminal activity versus controlling public health-related behaviors. Yet, although the rural context created tensions for law enforcement in balancing health and safety, and many communities faced structural lacks that challenged their abilities to respond to and manage an outbreak, rural justice leaders often argued that rurality was also a strength in the fight against COVID-19. They chose to play up their connection to and support from rural communities, emphasizing their insider statuses and what they saw as the benefits of rurality. Many pushed back against the narrative of rural lack, and instead insisted that small populations, high cohesion, close working relationships with health care providers, and physical space helped them to navigate the pandemic more effectively.

The sheriffs and local leaders in our study focused mostly not on rural challenges but on the strengths of rural communities when communicating with us and with the public regarding the virus and their communities’ responses. In addition to portraying themselves as tough on crime, many sheriffs’ Facebook pages highlighted their pride in the support they received from local communities. Many posted pictures of gifts they received from community members including care packages, cookies, and cards, or displays of support such as the flying of “Thin Blue Line” flags by local businesses. They posted pictures of newly hired corrections officers and deputies, and highlighted points of local and community pride. They spoke to us of the buy-in they received from local residents, businesses, and schools, and of the importance of retaining public trust.

They also spoke publicly about the ways in which rural communities were exceptional and uniquely insulated from the pandemic. In a county concerned about the negative impacts of stay-at-home orders on the local labor market, leaders commented that the population was used to stockpiling and social distancing, and that physical distance between homes helped to keep the virus at bay (Quinlan, 2020). The idea that rural lifestyles made these communities more resilient in the face of the pandemic was repeated by multiple sheriffs in our study, including the Facebook comment from the sheriff who posted this statement in an attempt to push back against the Governor’s orders:

[Our] County does not fit into the same mold as urban areas and we are able to adjust our habits, as we all already have, and continue to control the spread of the virus while also being able to enjoy ourselves and continue to provide for our families. We already reside in an area that lends itself to social distancing efforts by our rural geography and lifestyle. I feel that it is time to transition to strategies that fit the communities through local controls.

Multiple sheriffs also spoke of the benefits of being a “close-knit, tight community [where] everybody supports everybody, and everybody sticks together” (Quinlan, 2020). One sheriff discussed with us the importance of being a cohesive community where people want to do the right things for the right reasons. In our conversation with a rural lieutenant, he similarly talked about the importance of being tightly knit and working together, providing examples like local distilleries switching to making hand sanitizer for the community. He suggested that rural exceptionalism even included the local jails, which he argued could more easily support social distancing than large urban ones and could be nimbler and quickly adjust to the new realities of pandemic life. Although sheriffs acknowledged some of the structural challenges they faced, including the struggles to compete for PPE in the early days of the pandemic, publicly they focused on the advantages that rural communities had and downplayed their vulnerabilities both in terms of protecting health and in terms of protecting public safety. As the pandemic forced them to choose between public health that was seen as an urban and liberal issue, and public safety that was seen as a rural issue and one that safeguarded freedom, they made clear which side they were on. They chose informal controls to safeguard health, suggesting again and again that rural communities could be relied on to take care of themselves in this arena and that law enforcement did not need to intervene. And they reserved their punitive power for those seen by their constituents as an explicit threat to safety versus health, even when it meant outsized punishments and inadequate protections for those caught in rural justice systems. Although these measures, by and large, did not protect them from the ravages of the pandemic, they did protect the authority and legitimacy of rural law enforcement.

## Discussion and Conclusion

The experiences of counties in our study paint a complex picture of the pandemic’s impacts on rural Washington in terms of health and safety. While rural communities did have advantages including social cohesion and a context of physical space and low density, their cultural and structural contexts presented serious challenges to their abilities to influence public health. In some ways, rural isolation worked as a protective factor, including buying counties time to learn about the pandemic and begin to prepare before they were faced with cases either in their communities or within their jails. Our most isolated counties had few cases even months into the pandemic, while others were simply slow to experience the deadly wave. This time allowed local departments to put plans in place and to better understand the pandemic before they were faced with its harsh realities. Small-town settings also provided support for local law enforcement—if trust was maintained. As one sheriff told us,

Everything is personal in a community that is so small . . . You have to be a member of the community you police, and that way they don’t see you as them, they see you as a member of the us, of their own community, and people who are doing a job.

But these same small-town dynamics also tied sheriffs’ hands in multiple ways when it came to the pandemic and introduced both structural and cultural barriers to effectively combatting the outbreak. Rural jails struggled to equip themselves and lacked necessary medical facilities and personnel for when the virus did hit. It meant they were constrained in their abilities to limit their jail populations and keep space available, and that the only way to handle new jail admissions during the pandemic was to put people in solitary confinement sometimes for weeks at a time. Virtual courts were less accessible to some and courtroom delays and trial cancellations meant lengthier stays for many in jail.

And most important, the need for elected sheriffs to retain local trust and to be seen as “a member of the us” often restricted their abilities to support and enforce numerous safety restrictions, from stay-at-home mandates, to limiting in-person services, to mask mandates. As this article has described, rural law enforcement throughout our region walked a fine line between enforcement of state mandates and retention of public trust within conservative rural communities. Given the small size of their jurisdictions and their high visibility within the communities, they could not easily afford to take unpopular stances. Thus, rural sheriff’s departments repeatedly chose strategies that limited their abilities to protect populations from the disease, in favor of seeming tough on crime and supportive of personal liberty.

And thus, it was that by mid-summer and into autumn, counties throughout rural Washington were facing rapid rises in coronavirus caseloads, and many were beginning to experience strains on their inadequate medical systems as well as to their limited economies and labor markets (Leatherby, 2020). Despite the rhetoric of rural exceptionalism, they ultimately were not saved by their distanced lifestyles or physical space; or rather these assets did not protect them from the deleterious impacts of refusals to social distance, stay at home, or wear masks (Stone, 2020). While toughness on crime helped sheriff’s departments to reinforce their credentials and identities as locals, community members, and insiders, it left them a limited range of tools to fight for their law-abiding constituents’ health during the pandemic. Despite the cohesion and community concern that supposedly were rural communities’ main assets, cultural and political stances that prioritized individual choice, freedom, and liberty over common and community good remained their weaknesses.

Thus, as the pandemic progressed from the heat and sun of summer into winter’s dark, damp, and cold, rural communities throughout Eastern and Central Washington began to suffer devastating outbreaks, and even the most remote places were ravaged (O’Neill, 2021). Yet, stances toward public safety and public health were mostly unaltered. Sheriffs continued to focus their Facebook posts on nonpandemic-related issues, except when the disease infiltrated their jails and/or they were forced once again by state ordinances to limit services such as visitation or fingerprinting. Community responses to these posts were mixed, including many who supported sheriffs and acknowledged the virus’ severity but also many who challenged the reality of the pandemic and the necessity of protective measures. The most critical posts lashed out at sheriffs for attempting to control the virus, even wishing them ill and/or threatening them with replacement. A mid-December, 2020 sheriff’s post describing the jurisdiction’s reactions and precautions after discovering a COVID-19 case at the local jail provoked the following response:

Ever heard of “herd immunity”? Stop falling for the fear tactics! This is nothing more than a different strain of the seasonal flu! Unless you are FOR the one world order, which it sounds like you are. Grow a set and . . . let EVERYONE open back up! I see a new sheriff coming for [this] County!!!

The rare sheriffs’ posts on COVID-19, whether about local cases or new state-mandated restrictions, prompted many dozens of angry comments. Sheriffs responded by reiterating again their stances of nonenforcement of public health measures.

As we have shown, among the biggest rural constraints to adequately addressing the pandemic through behavioral modifications were the prevalence of cultural norms that rejected both the science and the politicized implications of those behaviors. The rural West has long been recognized for specific cultural understandings that focus on independence and individualism (Massip, 2012; Parker, 2011; Sherman, 2009), which can undermine the potential for collective efficacy. In the case of the pandemic, this research finds that public health is not effectively pursued through solely individualistic actions and agendas, which too often ignore the collective good in favor of personal preferences and desires. For rural law enforcement this cultural stance presented an unwinnable dilemma. In order to retain legitimacy and acceptance sheriffs and their staffs needed to respect their communities’ symbolic boundaries (Lamont & Fournier, 1992; Lamont & Molnar, 2002) between a law-abiding “us” that was seen as deserving unlimited autonomy and freedom, and an imagined other that was dangerous, “criminal,” and in need of strict policing. This limiting vision of individualistic freedom that explicitly rejected collective action with regard to the virus meant that rural sheriffs had little choice other than to sacrifice public health while focusing shortsightedly on public safety.

## Notes

1. The Centers for Disease Control and Prevention’s Social Vulnerability Index includes “a number of factors, including poverty, lack of access to transportation, and crowded housing [that] may weaken a community’s ability to prevent human suffering and financial loss in a disaster.” (Agency for Toxic Substances and Disease Registry, 2018)

2. Both the qualitative and quantitative studies have been approved by the Washington State University Institutional Review Board.

3. In one case, data were published biweekly, rather than daily, and did not include intake or release dates nor reason for incarceration/release.

4. Names and locations of network partners and data sources are withheld to protect confidentiality.

5. Posts are reproduced in the original language and grammar of the poster, except in cases where typos and grammatical issues create problems for understanding the posts. In these cases, minor edits have been made.

6. Options were limited by space constraints and aging jail facilities, by state laws and safety regulations for housing inmates, and personnel shortages in rural sheriff’s departments. Several of the counties in our study are in the process of planning and/or lobbying for building new, larger jail facilities; however, these processes are lengthy and will likely not be completed before the pandemic has ended.

7. The U.S. Census estimates that only about 0.4% of Washington homes lack telephone service. However, rural residents are often underserved when it comes to both Internet and cell phone access (Edelen, 2020; Ghajar et al., 2019; Wessels, 2013). In our study counties, between 15% and 25% of residents lacked access to broadband Internet (U.S. Census Bureau, 2019).

8. Names of individuals are all pseudonyms to protect confidentiality.

9. While some interview participants described benefitting from pandemic-related services like cash payments, unemployment insurance, and pandemic-related food and housing programs, many others complained of difficulties in accessing these types of benefits.
